# P-515. Three-Year Epidemiological Surveillance of Respiratory Syncytial Virus (RSV) Infections in Pediatric Patients at a Tertiary Care Center in Southern Puerto Rico

**DOI:** 10.1093/ofid/ofaf695.730

**Published:** 2026-01-11

**Authors:** Adanis C Bravo Cordero, Maria Jose Sanchez Muñiz, Yesabeli Condor, Vylma Velazquez Almodovar, Gabriela M Henriquez Luthje

**Affiliations:** San Juan Bautista School of Medicine, Caguas, PR; Universidad Central del Caribe, Bayamon, Puerto Rico; Children’s Hospital of Michigan, Detroid, Michigan; Saint Luke’s Episcopal Hospital/Ponce Health Science University., Ponce, Puerto Rico; Children's Mercy Hospital, Kansas City, Missouri

## Abstract

**Background:**

Respiratory Syncytial Virus (RSV) is a major cause of lower respiratory tract infections and hospitalizations in infants, especially those born prematurely. It is the second leading cause of infant mortality worldwide. Although the CDC recommends monoclonal antibody prophylaxis for high-risk groups, access in Puerto Rico remains limited.

From 2017 to 2023, five RSV epidemic seasons were identified in the U.S., with a notable absence in 2020, likely due to COVID-19 mitigation measures. RSV activity resurged in 2022–2023, accompanied by increased co-infections with SARS-CoV-2 and influenza, complicating pediatric care. However, limited RSV surveillance in Puerto Rico hinders a comprehensive understanding of local trends.

This study examines RSV incidence and seasonal patterns in pediatric patients at a major tertiary hospital in southern Puerto Rico, focusing on the impact of viral co-infections.

**Figure:**
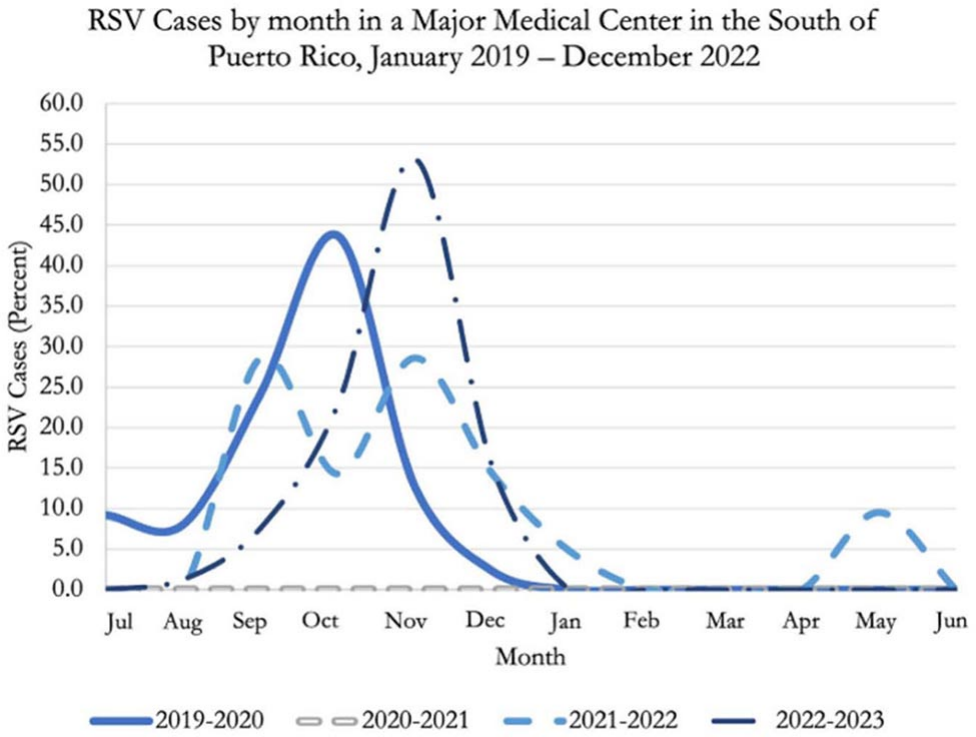
Monthly distribution of the percentage of respiratory syncytial virus (RSV) infection cases between 2019 and 2023 in the South of Puerto Rico Each line represents a calendar year, allowing for observation of seasonal variations and changes in year-to-year incidence. Under normal conditions, case peaks are recorded between October and December.

**Figure:**
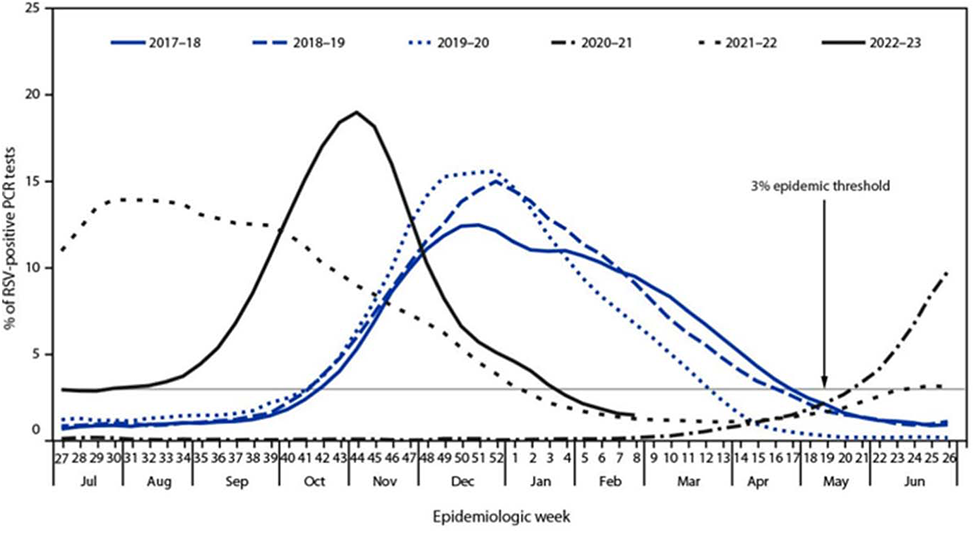
Monthly distribution of the percentage of positive PCR tests for RSV in the United States between July 2017 and February 2023, based on data from the National Respiratory and Enteric Virus Surveillance System. Each line represents an epidemiological year. In typical seasons, peaks in positivity are observed between October and December. This graph is used as a reference to compare the seasonal trends observed in our study. Source: Centers for Disease Control and Prevention (CDC), 2023. Retrieved from https://www.cdc.gov/mmwr/volumes/72/wr/mm7214a1.htm

**Methods:**

A retrospective observational study, approved by the IRB, was conducted from 2019 to 2022 to evaluate RSV-positive cases in the pediatric population. A total of 197 cases met the inclusion criteria and were analyzed.

**Results:**

In 2019, RSV incidence per 100 pediatric population (ages 0–5) was 4.5. No RSV cases were reported in 2020 due to COVID-19 restrictions. In 2021 and 2022, incidences of 0.7 and 1.5, respectively, were noted. Infants, often with comorbidities such as prematurity, accounted for 60% of admissions and were the most affected group. The study documented eight co-infections, including RSV and influenza in 2019, and a triple infection of COVID-19, RSV, and influenza in 2022.

**Conclusion:**

The study analyzed RSV trends, identifying an increase from September to December 2022, likely linked to relaxed COVID-19 regulations. Similar patterns were observed in the southern U.S. Contributing factors included reduced viral immunity among vulnerable groups and circulation of pre-existing RSV strains. RSV remains a significant public health concern, underscoring the urgent need for stronger prevention efforts. In Puerto Rico, national implementation of the monoclonal antibody nirsevimab would offer an effective prophylactic option to prevent severe infections and reduce the healthcare burden associated with RSV hospitalizations.

**Disclosures:**

All Authors: No reported disclosures

